# A sustainable approach to cathode delamination using a green solvent†

**DOI:** 10.1039/d1ra04922d

**Published:** 2021-08-11

**Authors:** Onurcan Buken, Kayla Mancini, Amrita Sarkar

**Affiliations:** Department of Chemistry & Biochemistry, Montclair State University NJ 07043 USA sarkara@montclair.edu

## Abstract

Designing an environment-friendly delamination process for an end-of-life (EoL) composite cathode is a crucial step in direct cathode recycling. In this study, the green solvent dimethyl isosorbide (DMI) is explored to extract cathode active materials (AMs) from the Al current collector *via* dissolving the polyvinylidene fluoride (PVDF) binder. Mechanistic insight suggests that binder removal from the Al substrate proceeds *via* reducing polymer interchain interaction through DMI penetrating into the PVDF crystalline region. Polymer–solvent interaction may increase *via* establishing hydrogen bond between PVDF and DMI, which facilitates binder removal. Analytical characterizations including ^1^H NMR, FTIR, XRD and SEM-EDS reveal that the molecular, micro, and crystal structures of the recovered cathode AMs, PVDF and Al foil are preserved. This finding is expected to provide a replacement for the toxic organic solvent *N*-methylpyrrolidone (NMP) and offers an effective, ecofriendly, and sustainable direct cathode recycling approach for spent Li-ion batteries.

## Abstract 2

A green solvent-based methodology was developed for delaminating cathode active materials from aluminium current collectors in end-of-life Li-ion batteries.

## Graphical abstract

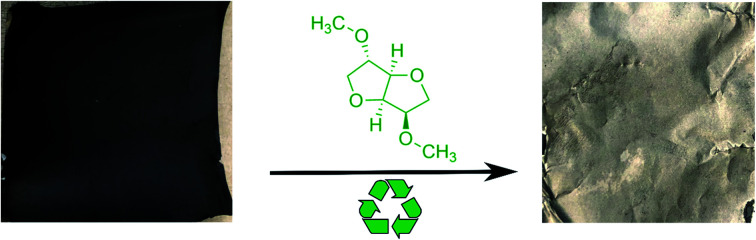

## 1. Introduction

Global efforts to accelerate the use of zero emission electric vehicles (EVs) with the aim of increased energy security and improved air quality has resulted in employing Li-ion batteries (LIBs) as a power source.^1^ However, their extensive use causes accumulation of enormous battery waste at the end-of their lives (EoL) which introduces a serious waste-management challenge. A thorough look at the important components of batteries reveals that the cathode is a combination of active materials (AMs) (*e.g.*, Ni, Co) and conductive carbon that adheres to a current collector aluminum (Al) substrate with a polymeric binder of polyvinylidene fluoride (PVDF). It is a waste of valuable resources if these components are not extracted from spent batteries.^1–3^ In view of their crucial usefulness, recycling of consumable rechargeable batteries at their EoL is a necessity to maintain their sustainability and economic benefits, mitigate the safety hazards associated with their disposal, and avoid extraction of Co and Ni minerals.^3–5^ State of the art technology related to the recycling of spent LIBs typically focuses on a combination of physical and chemical approaches including dismantling, shredding, thermal pretreatment, mechano-chemical, pyro, and hydrometallurgical processes.^2,6–13^ Although many recycling technologies have been industrialized,^2–4^ easy delamination of cathode AMs from the Al substrate without using high temperatures and toxic chemicals remains elusive. Recently, direct recycling of cathode materials has been proposed as a promising pathway where recycled materials with minimum changes to the crystal structures and surface patterns are incorporated in a new electrode composite film.^14–16^ During this operation, recovery of cathode AMs through the solvent dissolution method was found to be an efficient and safe route.^16,17^ This approach involves a range of different solvents which can effectively dissolve the PVDF binder at lower temperatures (*e.g.*, ≤100 °C), and accelerates the removal of AMs. Organic solvents including *N*-methyl pyrrolidone (NMP), *N*,*N*-dimethyl formamide (DMF), and *N*,*N*-dimethylacetamide (DMAC) are widely used in separating AMs.^18–25^ NMP is the best choice of solvent as it disrupts the strong interchain interaction in the PVDF crystalline region,^22^ weakens the polymer attachment in the Al substrate, and eliminates PVDF without disturbing its molecular and micro structures.^25^ Despite great success, the use of NMP increases the recycling cost^2^ and often causes slow biodegradability and high risks to the ecological environment and human health.^16^ Furthermore NMP is classified as an “undesired organic solvent” and not in agreement with the 12 principles of green chemistry.^26–28^ While toxic organic solvents have been explored in the literature, an advanced cathode delamination technique involving biobased and environmentally benign reagents remains elusive and of great interest to find with easy availability and low cost.^16,29,30^

Recently, the use of dimethyl isosorbide (DMI) was reported in fabricating a PVDF based ultra and microfiltration membrane, demonstrating a potential solvent alternative to NMP.^31^ DMI is a sugar based solvent, known as one of the top 10 bioderived chemicals,^32^ and commercially used in pharmaceutical additives and personal care products due to its non-toxic nature and water solubility.^33^ At present, isosorbide production has attracted widespread attention and is anticipated to reach US $324.6 million in the next few years.^34,35^ Several facile synthetic strategies for DMI have been reported^33,36,37^ to increase its availability at a cheaper price. The close proximity of the Hildebrand solubility parameters (*δ*_T_) of DMI to PVDF suggests its suitability as a dissolution solvent.^38^ Likewise, the Hansen solubility parameters (HSPs) for DMI demonstrate a similar hydrogen-bonding potential (*δ*_h_) to NMP. PVDF dissolution in DMI can be explained considering the hydrogen bonding established between the hydrogen and fluorine atoms in PVDF and the furanic oxygen, hydrogen, and methoxy groups in DMI.^30,36,39^ Additionally, the affinity and relative energy difference (RED) parameter is <1 (Table 1), which further confirms PVDF miscibility in the chosen solvent.^40–42^ In this study, DMI is successfully applied in cathode delamination, which results in selective removal of active materials (AMs) from the Al substrate *via* dissolving PVDF. A complete characterization of recovered materials was performed through nuclear magnetic resonance (NMR), Fourier transform infrared spectroscopy (FTIR), scanning electron microscopy (SEM), energy dispersive X-ray spectroscopy (EDS), and powder X-ray diffraction (XRD). The dissolution mechanism of PVDF in DMI is discussed here. To the best of our knowledge, this is the first report of DMI-based cathode delamination in a sustainable fashion as a greener alternative to NMP.

**Table 1:** Hildebrand and Hansen solubility parameters (HSPs)

Polymer/solvent	Hildebrand *δ*_T_ (MPA)^1/2^	HSPs *δ*_d_ (MPA)^1/2^	HSPs *δ*_p_ (MPA)^1/2^	HSPs *δ*_h_ (MPA)^1/2^	RED_PVDF_	Toxicity
PVDF^37,43^	24.2	17.2	12.5	9.2	—	—
NMP^37,43^	23.0	18.0	12.3	7.2	0.4	Hazardous
DMI^44^	20.4	17.6	7.1	7.5	0.9	Green

## 2. Materials and methods

### 2.1. Materials

Discharged and dismantled EoL Samsung battery cathodes were kindly supplied by Wesley Chang, Princeton University. Lithium cobalt(iii) oxide (LiCoO_2_, LCO), lithium manganese oxide (LiMn_2_O_4_, EQ-Lib-LMO), and lithium nickel manganese cobalt oxide (LiNi_*x*_Mn_*y*_Co_*z*_O_2_, *x* : *y* : *z* = 1 : 1 : 1, NMC 111) were purchased from MTI Corporation and calcined at 600 °C in air prior to use. Super P conductive carbon black (C45), graphite powder (carbon content >99.5%), and poly(vinylidene fluoride) binder (PVDF, >99.5%) were purchased from MTI Corporation and used as received. Isosorbide dimethyl ether (DMI, Sigma Aldrich, >99%), *N*-methyl-2-pyrrolidine (NMP, anhydrous, >99%, Sigma-Aldrich), dimethyl carbonate (DMC, Sigma Aldrich, >99%), 1 M lithium hexafluorophosphate (LiPF_6_) solution in ethyl carbonate (EC), dimethyl carbonate (DMC) mixture (EC : DMC 1 : 1, v/v) (LP30, Sigma-Aldrich), dimethyl sulfoxide-d_6_ (DMSO-d_6_, 99.9% atom D, Cambridge Isotope Laboratories), and deuterated water (D_2_O, 99.9% atom D, Cambridge Isotope Laboratories) were commercially purchased, stored at ambient conditions, and used without further purification. Diethyl ether (anhydrous, ACS reagent, ACROS Organics) was stored at 2–8 °C.

### 2.2. Commercial battery disassembly

Two cells including 220018-4-6-109 and 220023-2-4-120 that had undergone a few months of long-term cycling were disassembled and used in this study to investigate the role of DMI in the recovery of the cathode component. Post-mortem analysis with EDS indicates that the cathode might be mixed or blended type (*vide infra*). However, the battery packs did not contain information about the chemistry of the cathode; details are reported in Table S1.† Cell 220018-4-6-109 had undergone 811 cycles at a rate of 2C under constant current constant voltage (CCCV) charge and constant current discharge. Likewise, cell 220023-2-4-120 had undergone 1120 cycles at a rate of 1C under constant current (CC) charge and discharge. After the cycling experiments, the cells were stored at rest for a year before disassembly. Immediately before disassembly, the cells were fully discharged to minimize the risk of explosion and transferred into the Ar-filled glovebox. An electric Dremel tool was used at a medium power along the edge of the cells to cut open the cap and then along the side to unfurl the cylindrical container. The electrodes were unrolled and the cathode side was peeled off, washed, and used in subsequent studies.

### 2.3. Characterizations

^1^H NMR spectra were recorded on a Bruker 400 spectrometer at room temperature and analyzed by MNOVA software. ^1^H NMR chemical shift values (*δ*) were calibrated using the solvent peak (from the residual solvent protons, *e.g.*, 2.51 ppm for DMSO-d_6_ and 4.79 ppm for D_2_O). For all measurements, a scan number of 64 is used. Fourier transform infrared spectroscopy analysis (FT-IR) of the commercially purchased and recovered PVDF was carried out on a PerkinElmer Spectrum Two FT-IR spectrometer. Measurements were carried out in the range of 3000–650 cm^−1^ with a scan number of 32. Top-view SEM images of the purchased and recovered cathode AMs microstructures were acquired using Hitachi S-3400N SEM at the Microscopy and Microanalysis Research Laboratory (MMRL), Montclair State University. Samples were mounted on aluminum stub using carbon adhesive and imaged normal to the planar surface. An acceleration voltage of 15 keV and a secondary electron detector were used. Working distance was maintained at ∼6–10 mm. The elemental composition of the specimens before and after DMI based cathode delamination was examined by a Bruker Xflash EDS. Both qualitative and quantitative analyses were performed. Diffraction patterns of recovered cathode AMs were collected on a Rigaku MiniFlex 6G Powder diffractometer equipped with a 600 W Cu Target X-ray source. XRD patterns were collected in the range of scattering angles 2*θ* of 10°–90° by steps of 0.010° and a scanning rate of 5° min^−1^. Data analysis was performed using software Rigaku Smart Studio II. Phase identifications were performed referencing to the crystallography open database (COD).

### 2.4. Lab scale cathode delamination approach for Samsung 220018-4-6-109

**Scheme 1:**
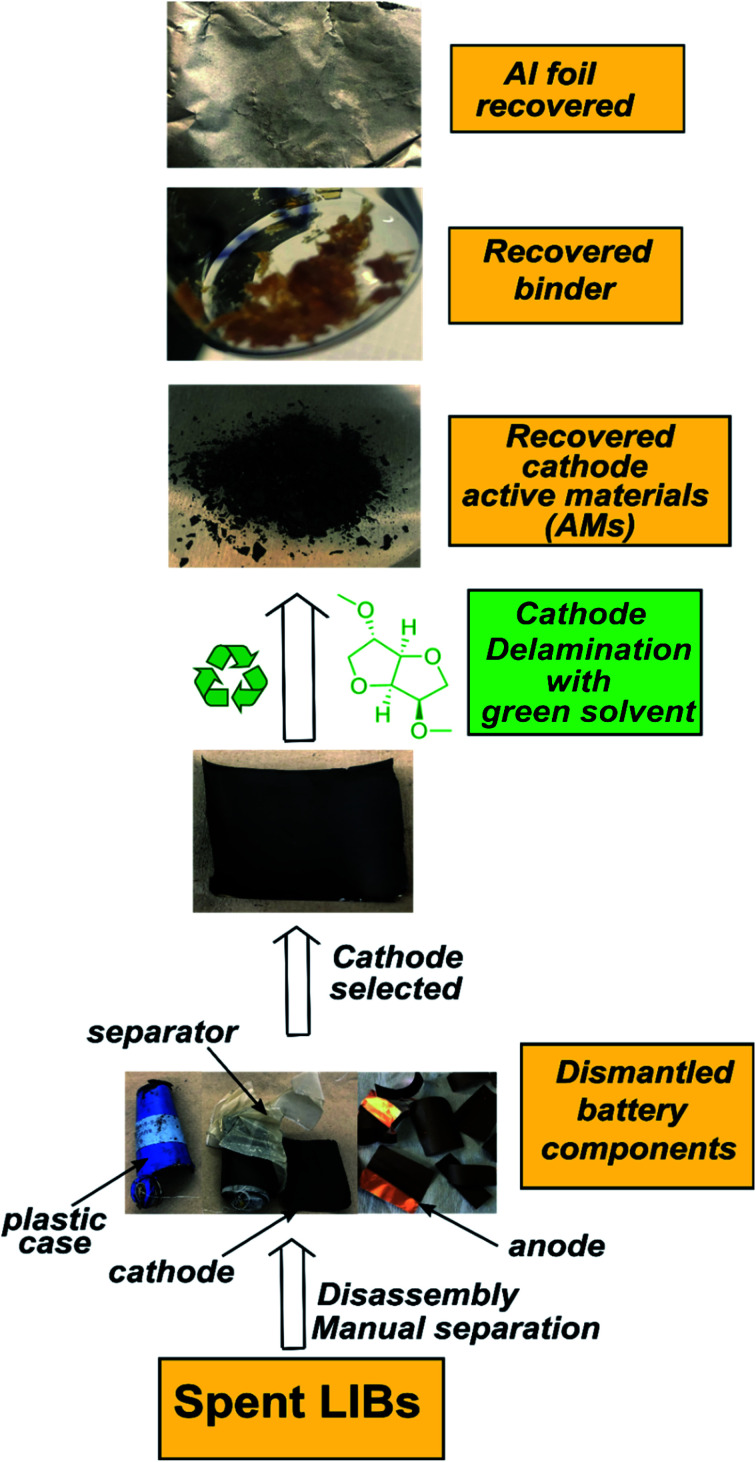
The outline for the DMI based cathode delamination process to recover AMs, polymer binder, and current collector Al foil from the EoL Samsung battery.

Here we report a DMI-based cathode delamination technique for EoL Samsung battery and recovered cathode AMs, PVDF binder and Al current collector, as outlined in Scheme 1. To optimize the delamination procedure a range of experimental conditions were tested, detailed in Section 1, Section 2, Tables S2 and S4 in ESI.† We note that while most home-made cathode composites were dissolved in DMI only after 30 minutes heating at 150 °C, the process had to be extended to at least 5 h for those cathodes that were harvested from EoL Samsung batteries. Extended time is needed possibly due to differences in cathode manufacturing, or electrochemical treatment. Having identified the optimal experimental conditions, a solid to liquid (S/L) ratio of 1 : 40 g : mL was selected. A reaction temperature of 150 °C for 5 h allowed facilitated delamination of cathode AMs from the Al substrate in Samsung 220018-4-6-109. The detailed experiment procedure is described in Fig. S1.† 5 g discharged and disassembled cathode films was washed twice with 10 mL dimethyl carbonate to wash off the residue electrolyte. Electrodes were cut into approximately 5 cm × 5 cm pieces and immersed in 200 mL DMI in a round bottom flask with continuous mechanical stirring, set into a preheated oil bath at 150 °C. Next, the dark brown colored solution was cooled to room temperature and filtered. Al substrates along AMs were separated from the liquid DMI. Please note that the cathode AMs detached partially from the Al foil during the course of reaction. However, the AMs fully recovered only after manual scraping of the Al substrate.^23,30^ Peel-off or delamination efficiency could be improved by ultrasonication.^18,45^ Recovered AMs were filtered, dispersed in 10 mL hot DMI followed by 10–30 minutes sonication, washed with 10 mL diethyl ether, and dried at 60 °C under vacuum for 2 days. This set of samples was labelled “*as recovered*” cathode AMs. Likewise, the Al substrates were washed twice, each with 10 mL DMI and diethyl ether, respectively, followed by drying under vacuum at 60 °C for 2 days. Liquid DMI was precipitated in 5× chilled anhydrous diethyl ether set into a liquid nitrogen bath for recovering the binder. The top, clear liquid portion was carefully discarded and the remaining viscous solution was evaporated under vacuum to obtain a yellowish-brown binder. The recovered binder texture and color was different than the commercially purchased PVDF and could be attributed to various factors including the presence of impurities or by-products developed during electrochemical cycling, residual carbon content, or minor impurities present in DMI.^25^

## 3. Results & discussion

### 3.1. Cathode delamination *via* the green solvent dimethyl isosorbide (DMI): proof of concept

**Fig. 1:**
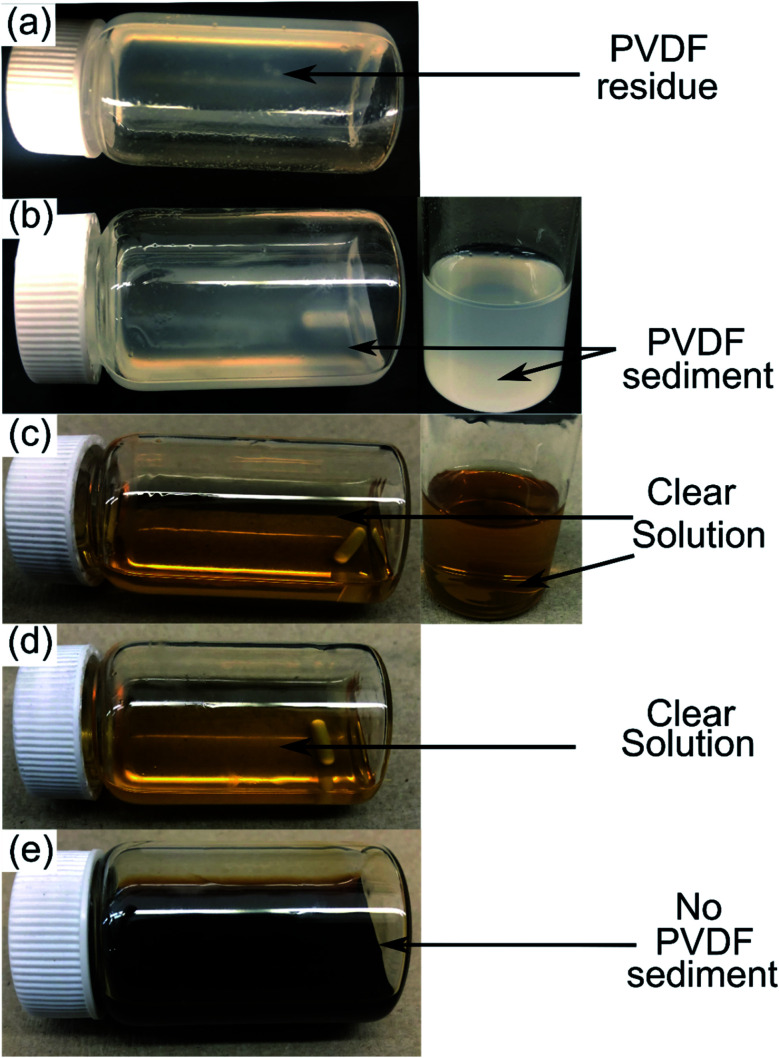
10 wt% PVDF solution in DMI observed at 25 °C (a), heated at 60 °C for 8 h (b) and 150 °C for 8 h (c). Dissolved PVDF solution was cooled and stored at ambient conditions for 1 day, as shown in (d). The DMI based cathode delamination technique was applied on EoL Samsung 220018-4-6-109 at 150 °C and results in a dark but clear solution, as shown in (e).

PVDF is a semicrystalline polymer and known to be dissolved at room temperature by few organic solvents including NMP, DMF and DMAC. These solvents are capable of disrupting the PVDF interchain interaction at 25 °C (ref. 22) and result in forming clear solutions. Conversely, a solubility test conducted in DMI at room temperature leads to forming a viscous solution with traces of precipitating PVDF. Upon raising the temperature to 150 °C, rapid solubilization occurs, which suggests reduction of polymer interchain interaction *via* DMI penetrating into the PVDF crystalline region. Dissolved PVDF remains in the solution after prolonged storage (1 month) at room temperature and never precipitated. This finding is consistent with the characteristics of a “transition state solvent”.^30,42^ Results of the dissolution tests conducted at different temperatures are shown in Fig. 1. Experimental findings on the excellent dissolution property of PVDF led us to investigate the role of DMI in cathode delamination. In order to assess the potentiality of DMI as a selective solvent for cathode delamination, a lab-scale process was designed and applied on homemade composite cathode film and two EoL Samsung 18650 cylindrical cells, as outlined in Scheme 1 and Fig. S1.† Recovery yields for each component collected from the homemade composite film and Samsung 220018-4-6-109 (cycled at 2C for 811 cycles at 40 °C prior to disassembly) are presented in Tables S4† and 2, respectively. Homemade composite films were made with known masses of ingredients (Section 2, ESI†) and % recovery yields for the mixture of AMs and conductive C, PVDF binder, and Al were calculated as 98, 77 and 100% respectively, as detailed in Table S4.† On the other direction, 4 g cathode AMs, 120 mg binder and 865 mg Al foils were recovered from the 5 g Samsung 220018-4-6-109 cathode. The % recovery for AMs + C collected from Samsung 220018-4-6-109 was estimated based on the total cathode mass, as the specific masses of each ingredient were not disclosed by the manufacturer. The % recovery for the binder was estimated considering a typical 3% binder population in commercial cells. The values are reported as 81 and 79% for the mixture of AMs + conductive C and binder, respectively, as detailed in Table 2. Recovered components were extensively characterized by a series of analytical techniques, *vide infra*. Cathode AMs were calcined at 600 °C in air prior to characterization and identified as “*calcined*”. Relatively poor recovery was achieved for Samsung 220023-2-4-120 (Table S3†), which was cycled at 1C for 1120 cycles at 25 °C prior to disassembly. Lower recovery could be attributed to the extended electrochemical cycling conditions that result in strong attachment of the AMs onto Al and decrease the solubility of the binder in DMI by forming a preventive layer. No improvement in the recovery was found when the same cathode was delaminated using the traditional solvent NMP (Fig. S2†).

**Table 2:** Recovery yields from a commercial Samsung 18650 cylindrical cell^a^

Composite cathode ID	Total cathode mass (g)	Mass of recovered cathode AM + conductive C (g) after DMI treatment	% recovery of cathode AM + conductive C	Mass of recovered binder (g) after DMI treatment	% recovery of binder	Mass of recovered Al substrate (g)
220018-4-6-109	5	4	81 ± 3^b^	0.120	79 ± 6^c^	0.865

a Masses of each recovered components were measured on an analytical balance.

b % yield of AMs with conductive carbon mixture was calculated with respect to the total mass of cathode, ignoring the mass of the binder and substrate, as the specific amounts of individual component in the original cathode was unknown. Thus % recovery for AMs + conductive C is calculated as: 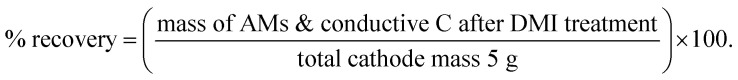

c Assuming this cell contains 3% binder (as typical commercial batteries consist of 2–4% binder), the % recovery was calculated as: 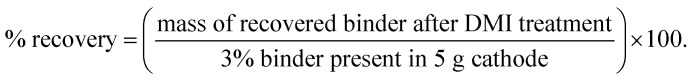 Error bars represent the standard deviation of the average for *N* = 3 measurements.

### 3.2. Characterization of delaminated cathode active materials (AMs)

**Fig. 2:**
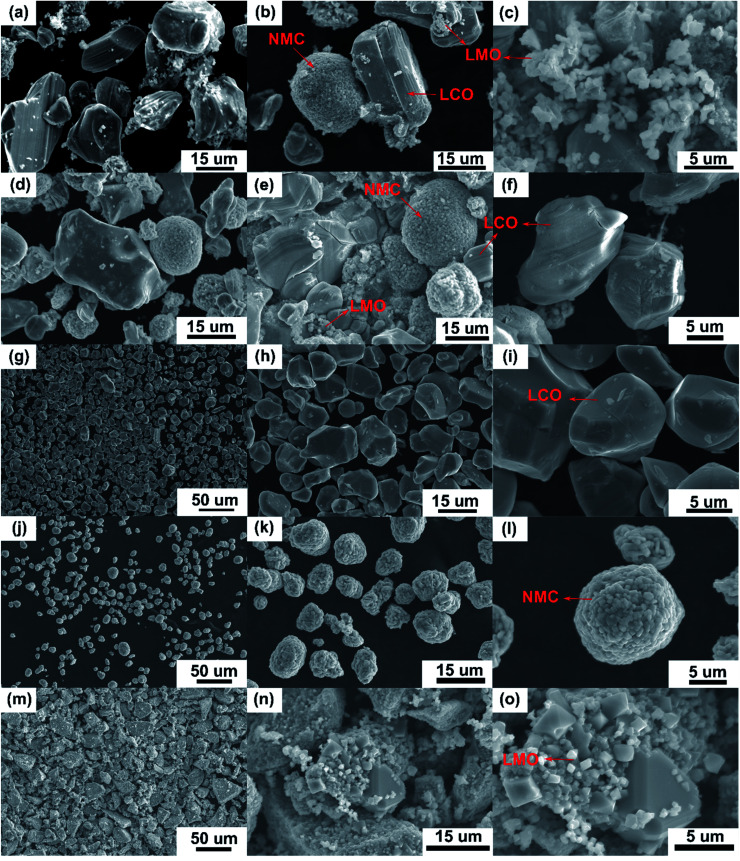
SEM micrographs of the “*as recovered*” (a–c) and “*calcined*” (d–f) samples from Samsung 220018-4-6-109 cathode suggest the electrode is mixed cathode or blended with possible compositions of LCO, NMC and LMO-type AMs (indicated by arrows). Surface images of these recovered AMs were compared to the commercially purchased LCO (g–i), NMC (1 : 1 : 1) (j–l) and LMO (m–o).

**Fig. 3:**
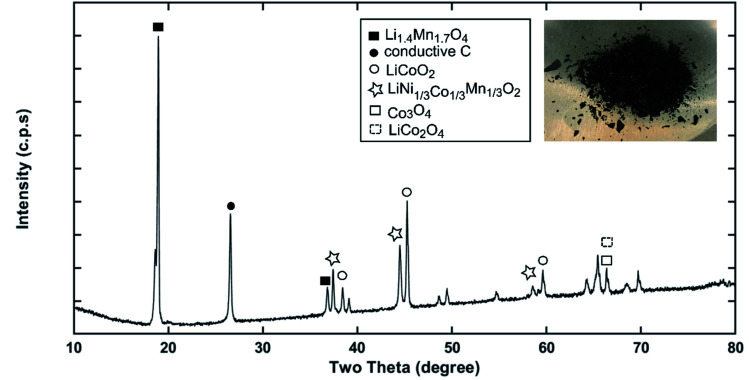
The XRD pattern of the “*as recovered*” material collected from Samsung 220018-4-6-109 suggests the possible cathode composition is mixed or blended.

Recycling cathode AMs are essential to consider due to the natural scarcity and growing demand of the critical elements (*e.g.*, Co, Ni) in the EV manufacturing industry.^46,47^ Thus, a direct cathode recycling approach is more desirable where the engineering value of the recovered AMs is maintained. It was our concern that DMI-based removal of binder may affect the surface and crystal structures of the AMs, hindering direct regeneration of materials for new cathode fabrication. To examine this, “*as recovered*” and “*calcined*” AMs were characterized by SEM (Fig. 2). Conductive carbon was found in the “*as recovered*” sample, which was not observed further in the “*calcined*” sample (Fig. S3†). Surface morphologies of both sets of samples were compared to the commercially purchased LiCoO_2_ (LCO), LiMn_2_O_4_ (LMO), and LiNi_0.33_Mn_0.33_Co_0.33_O_2_ (NMC 111). Visual inspections of the surface morphologies of the “*as recovered*” and “*calcined*” samples were consistent with the particle types of LMO, LCO and polycrystalline NMC (Fig. 2a–f and S4†), which suggests the electrode is mixed cathode or blended.^48,49^ Particle morphologies were found to be consistent with loosely bound aggregates (average size of 3.12 ± 0.9 μm) composed of well-defined single spinel crystals (average size of 0.98 ± 0.2 μm) of octahedral shaped LMO (Fig. 2c and e). Likewise the average sizes of the spherical NMC type secondary particles were found to be 11 μm which contain an agglomeration of numerous primary particles of 0.8 μm with standard deviations of 3 and 0.2 μm, respectively (Fig. 2b, e and S5†). Similarly, LCO type particles were found also with the average size range of 10.2 ± 4.3 μm (Fig. 2b and f). Additional compositional analysis of the cathode (before DMI treatment) separated from the EoL battery showed the presence of Co, Ni, and Mn (Fig. S6†). Energy dispersive X-ray spectroscopy (EDS) analysis detected P, which suggests the trace presence of LiPF_6_ electrolyte, which was not removed completely during the cathode washing step (Fig. S6†). The XRD pattern of the “*as recovered*” sample showed the peaks for LiCoO_2_ (LCO), Li_1.4_Mn_1.7_O_4_ (LMO), and LiCo_1/3_Ni_1/3_Mn_1/3_O_2_ (NMC) along with carbon additives (Fig. 3), which further suggests the mixed electrode compositions, consistent with SEM (Fig. 2). Thorough XRD analysis showed the presence of a weak reflection peak for Co_3_O_4_, which is known to be the performance-reducing LCO degradation product and is often generated during multiple electrochemical cycles in battery operation.^50,51^ However, the lack of information about the chemistry of the commercial battery complicated the identification of AMs and prevented a quantitative comparison of the changes in particle size before and after DMI treatment. To get insight into the role of DMI on the particles' morphologies and crystal structures, we repeated the DMI based delamination process on a homemade composite NMC 111 film with a known composition (Section 2, ESI†). Prior to delamination, the electrodes were immersed in LiPF_6_ electrolyte for a week and no electrochemical cycling experiments were conducted. Recovered AMs from the homemade composite cathode films were calcined and analyzed by SEM, EDS and XRD (Table S4, Fig. S7–S9†). Particle size analysis and surface morphologies of NMC 111 before (secondary particles average size = 9.87 ± 3.33 μm; primary particles average size = 1.15 ± 0.29 μm) and after (secondary particles average size = 9.15 ± 3.12 μm; primary particles average size = 1.17 ± 0.33 μm) solvent treatment showed no apparent changes. Likewise, diffraction peaks for the recovered NMC 111 confirmed all recognizable reflection planes, such as (003), (101), (104), (105), (107) and (113), which can be indexed as the layered oxide structure of the *R*3̄*m* space group. Specifically, distinct splitting of the (108), (110) and (006), (102) peaks confirmed the preservation of well-ordered hexagonal α-NaFeO_2_ crystal structures with no secondary phases.^52^ Altogether, SEM and XRD showed successful recovery of cathode AMs with durable stability, particle morphologies, and surface features that are useful during direct cathode recycling.

### 3.3. Identification and characterization of the recovered binder from Samsung 220018-4-6-109

**Fig. 4:**
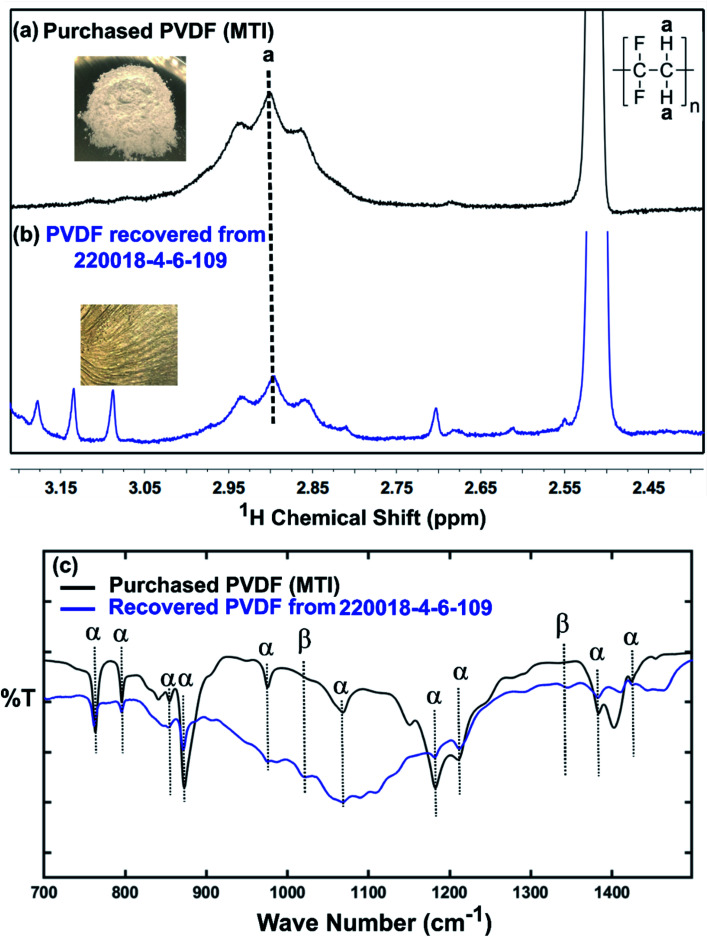
^1^H NMR spectra recorded in DMSO-d_6_ of commercially purchased (a) and recovered PVDF from EoL commercial Samsung battery (220018-4-6-109) (b). The strong (cut off) peak at 2.51 ppm is due to residual DMSO in the NMR solvent. Minor peaks at 3.087 and 3.134 ppm come from residual DMI, whereas the other minor peaks at 3.177, 2.809, and 2.612 ppm are attributed to unknown organics, recovered simultaneously. FTIR-ATR spectra of characteristic peaks for the α and β phases of the pristine and recovered PVDF shown in (c).

**Fig. 5:**
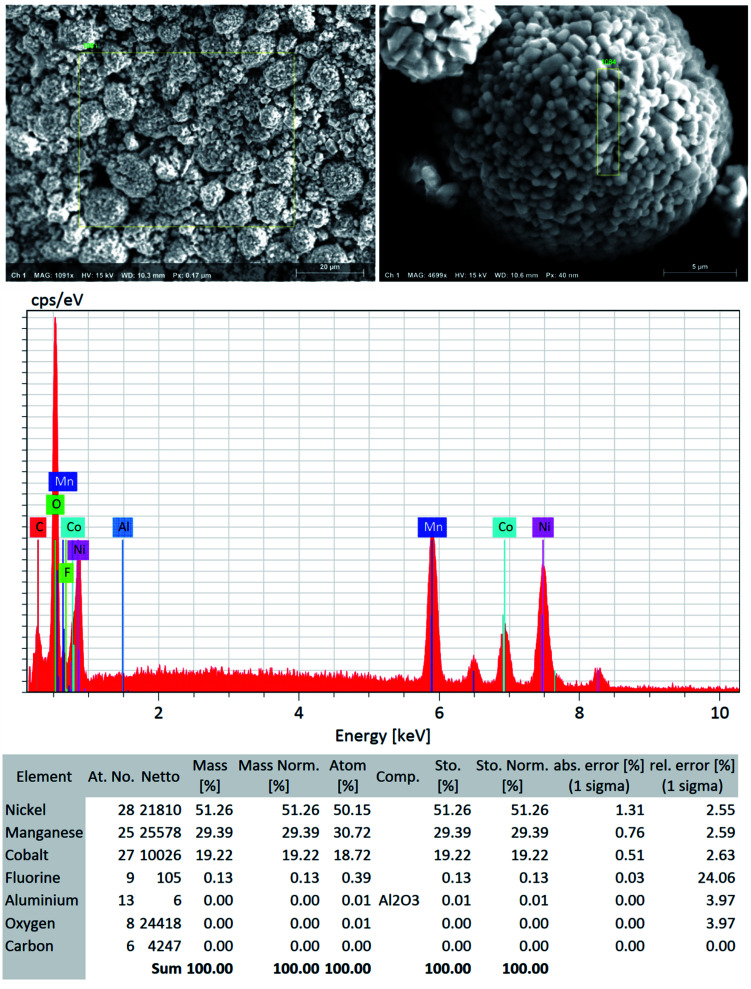
SEM-EDS quantitative analysis (the focused area on NMC type particle) shows low to no significant traces of F residue (∼0.1%) and Al particles (not detected) in the “*as recovered*” cathode AMs that confirms the efficient but benign mild nature of DMI based cathode delamination technique. Elemental composition suggests that the DMI-based approach removes PVDF successfully and usually does not corrode the Al current collector surface.

**Fig. 6:**
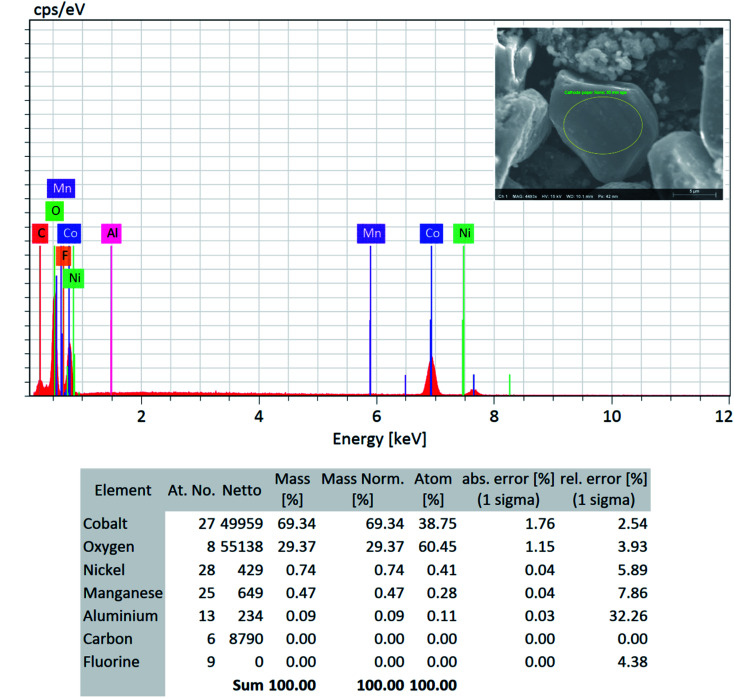
SEM-EDS quantitative analysis (focused area on the LCO type particle) on the “*as recovered*” cathode AMs detected no F and confirms its successful removal *via* the DMI based cathode delamination technique.

Although a very small percentage of polymer binder is needed to construct a cell and its cost is less than 10% over the entire battery fabrication, it is one of the crucial materials to improve the cell performance.^53^ PVDF is the most widely used cathode binder and often hinders the direct cathode recycling due to its capacity to hold the active materials tightly to the current collector.^54^ We herein expand the scope of the DMI-based method for dissolving and recovering binder from EoL batteries. Our developed method results in high recovery of PVDF binder (Tables 2 and S4†). Analysis of recovered binder with ^1^H NMR showed resonance at 2.895 ppm (Fig. 4a and b), which is assigned to head-to-tail (ht) bonding arrangements of vinylidene fluoride (VDF) units.^25^ Comparing the ^1^H NMR spectra of recovered binder to the commercially purchased PVDF not only identified the binder type, but also confirmed that no degradation happened at the molecular-level. Similarly, FTIR results (Fig. 4c) displayed the retention of crystalline forms α and β in recovered PVDF after DMI treatment. Non-polar α phases were identified at 762, 795, 974, 1212, 1382 and 1412 cm^−1^ in the pristine and recovered PVDF, which confirmed the predominance of kinetically controlled crystallization which was not altered after DMI treatment.^31,55^ Similarly, thermodynamically stable polar β-phase crystalline structures were found at 1068 and 1181 cm^−1^, which are expected to be observed when treated with polar solvent.^31,55^ Enormous battery waste likely participates in a large accumulation of plastics from binders, contributing to an already dismal plastic epidemic.^56,57^ Developing new strategies to recover and re-use polymer binder closes this loop and eliminates the creation of additional plastic waste in the environment. Taking all measurements into account, it can be concluded that PVDF binder is not only extracted selectively but also not associated with emission of PVDF degradation products, such as hydrogen fluoride and chlorofluorocarbon,^1,2,58^ and thus suitable for reuse in second battery fabrication.^25^ The residual F content from PVDF often leads to desquamation of the electrode particles and results in capacity fading.^59^ To ensure the complete removal of PVDF, “*as recovered*” mixed-cathode materials were examined by EDS, which clearly demonstrates low (Fig. 5) or no sign of F residue (Fig. 6). Thus, the DMI based robust delamination process paves the way for an advanced direction in cathode waste management strategies with the possibility of its reuse in subsequent new battery fabrication.

### 3.4. Influence of DMI on the removal of PVDF binder from the Al current collector

**Scheme 2:**
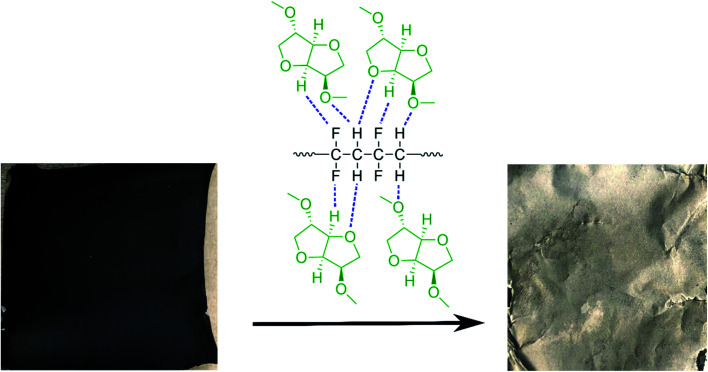
A schematic diagram of cathode delamination *via* the green solvent dimethyl isosorbide (DMI).

Taking all this information into consideration, we propose a plausible mechanism for the PVDF separation as presented in Scheme 2. Thermodynamic studies well predicted the solubility of PVDF in DMI, as shown in Table 1. PVDF adheres to the Al current collector *via* strong hydrogen bonding.^43^ Rapid solubilization of PVDF may occur *via* (i) reducing polymer interchain interaction through penetrating DMI into the PVDF crystalline region^22^ and (ii) enhancing the solvent–polymer interaction by establishing hydrogen bonding between the hydrogen and fluorine atoms in PVDF and the furanic oxygen, hydrogen, and methoxy groups in DMI.^30,36,39^ Separately, DMI may participate actively in hydrogen bond formation with the thin oxidation layer of aluminum oxide coating on the current collector that weakens the PVDF attachment in Al foil and facilitates PVDF removal.^60^

**Fig. 7:**
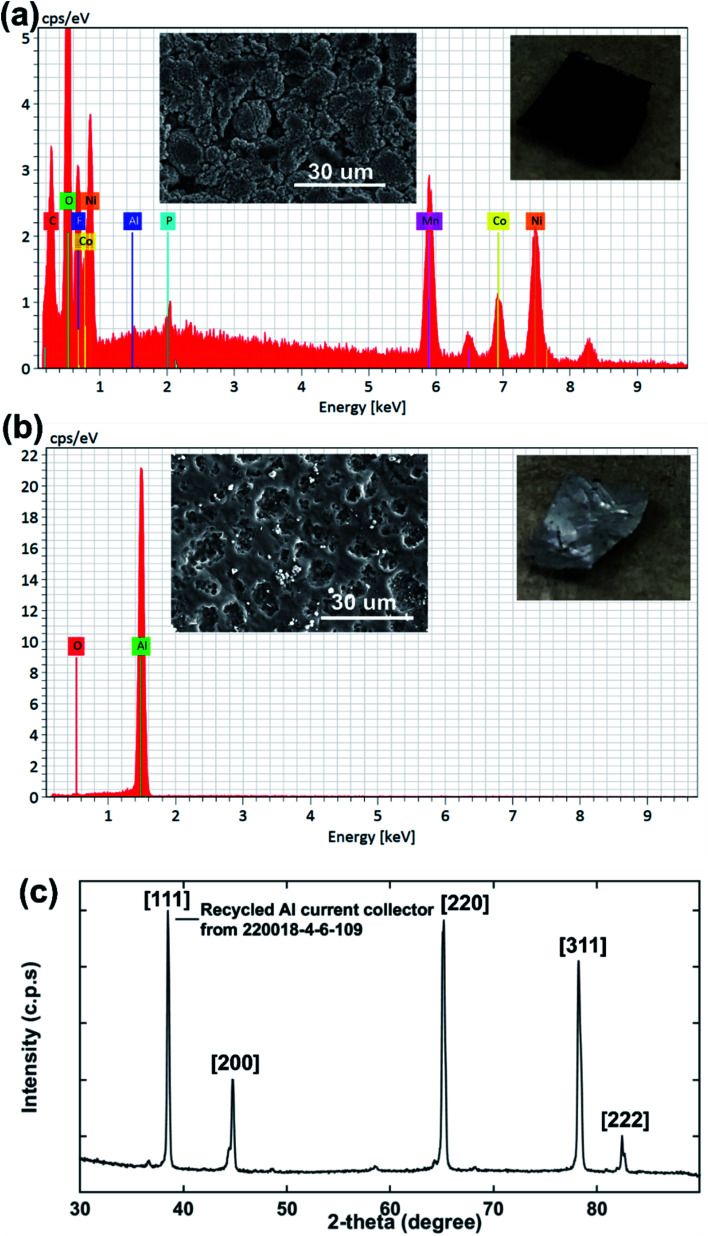
The surface morphology and elemental composition of cathode AMs adhering to the Al current collector before DMI treatment are shown in (a). SEM-EDS analysis shows that about 13 wt% fluorine content is estimated for the cathode electrode surface, which comes primarily from the PVDF binder. F is eliminated completely from Al after DMI treatment, as shown in (b). Elemental composition wt% is listed in Table 3. The XRD pattern for recovered Al foil (c) shows all relevant diffraction peaks.

In order to investigate the effectiveness of DMI in PVDF removal the Al substrate was recovered, washed with DMI and diethyl ether, dried under vacuum, and analyzed by SEM-EDS (Fig. 7). The disassembled cathode showed the presence of all active elements including Ni, Co, Mn, O along conductive C and Al substrate before the DMI treatment (Fig. 7a). The P signal was found in the same EDS spectra together with F, which suggests the presence of the LiPF_6_ electrolyte. Most importantly, about 13 wt% F content was detected due to the presence of PVDF binder, which completely disappeared after DMI operation (Fig. 7b), as summarized in Table 3. Moreover, all the transition metal elements and conductive C traces disappeared, which further confirms the successful delamination of AMs from the Al substrate through PVDF dissolution. However, the recovered Al substrate morphology showed visible surface damage (Fig. 7b), which possibly occurred during the manual scraping. However, no trace of Al particles was detected in the cathode AMs (Fig. 5), which typically migrate from corroded Al foils.^61^ Unaltered diffraction peaks for the Al substrate (Fig. 7c) further suggest that no significant chemical corrosion happened while encountering DMI. Thus, we conclude that our developed approach increases the total cathode delamination efficiency *via* recovering the additional component Al, which can reduce 33% total energy consumption when reused in EVs manufacturing industries.^62^ To maximize profitability, the DMI-based method was applied on the EoL anode (Samsung 18650 cylindrical cell 220023-2-4-120) and all the invaluable components including graphite, binder and copper (Cu) current collector were recovered successfully. Preliminary findings are described in Section 3, Table S1† and Fig. S10–S13 in ESI.† All together our solvent-based dissolution approach demonstrates the successful recovery of high-purity electrode materials, which can be reused in new cathode fabrication and may improve the process economics significantly.^63^ The manufacturing of new electrodes using recovered AMs, binder and Al foil and their electrochemical performance is currently under investigation. Future work will focus on the systematic investigation of the influence of battery ageing history and chemistry on the DMI based delamination efficiency.

**Table 3:** Elemental composition for the Al current collector (220018-4-6-109) by SEM-EDS analysis^a^

Conditions	Wt%_Ni_	Wt%_O_	Wt%_Mn_	Wt%_F_	Wt%_Co_	Wt%_Al_	Wt%_C_	Wt%_P_
Before DMI based cathode delamination	31	25.8	17.5	13	12.1	0.02	0.01	0.63
After DMI -based cathode delamination	—	1	—	—	—	99	—	—

a — not detected.

## 4. Conclusions

A Li ion battery recycling strategy must include a reduction plan for releasing toxic organic byproducts along with recycled material with sufficient purity. The present study focuses on the role of the green solvent DMI in the enhanced recovery of cathode AMs from the current collector *via* dissolving PVDF. This delamination technique not only recovers essential and additional components from EoL cathodes without compromising their quality but also mitigates the environmental challenges by eliminating the hazardous impact of NMP. We envision that this environment-friendly delamination approach will cause a step-change in Li-ion battery recycling and electronic waste circular economy.

## Conflicts of interest

There are no conflicts to declare.

## Supplementary Material

RA-011-D1RA04922D-s001
