# Supplementary material for: A sustainable approach to cathode delamination using a green solvent
Source: RSC Adv. 2021 Aug 11;11(44):27356–68. doi: 10.1039/d1ra04922d (PMC9037836; doi:10.1039/d1ra04922d)
Supplement: RA-011-D1RA04922D-s001 [file RA-011-D1RA04922D-s001.pdf]

Electronic Supplementary Material (ESI) for RSC Advances.
This journal is © The Royal Society of Chemistry 2021

Supporting Information

Sustainable Approach to Cathode Delamination Using A Green Solvent

Onurcan Buken, Kayla Mancini, Amrita Sarkar*

Department of Chemistry & Biochemistry, Montclair State University, NJ 07043
*Corresponding Author
Email: sarkara@montclair.edu

Table of Contents

Table S1. Details of commercial Samsung 18650 cylindrical cells……………………..…….S2

Section 1. Parameter optimization for DMI-based cathode delamination…………………..S2

Table S2. Summary of optimized parameters for DMI-based cathode delamination….…..S3

Fig. S1. Cathode delamination of Samsung 220018-4-6-109 using DMI………..………..…..S4

Table S3. Recovery of cathode components from battery 220023-2-4-120………………….S5

Fig. S2. Recovered Al substrate from EoL Samsung 220023-2-4-120……………………….S5

Fig. S3. SEM image of "*as recovered*" and "*calcined*" cathode………………..……….….S6

Fig. S4. SEM image of "*as recovered*" AMs shows possible cathode compositions………...S7

Fig. S5. SEM image of "*as recovered*" NMC primary and secondary particles……………..S7

Fig. S6. SEM-EDS analysis of cathode from Samsung 220018-4-6-109…………….…..…S8

Section 2. Homemade composite cathode film fabrication and delamination……..……….S9

Table S4. Recovery of cathode components from homemade composite film……………...S9

Fig. S7. SEM image of NMC 111 delaminated from homemade film…….……………..…S10

Fig. S8. EDS analysis of NMC 111 delaminated and calcined…….………………………..S11

Fig. S9. XRD of NMC 111 delaminated from homemade film……………………..…..…S12

Section 3. Lab scale anode delamination for Samsung 220023-2-4-120..……………………S12

Fig. S10. SEM micrograph of "*as recovered*" anode materials…………………………..…..S13

Fig. S11. SEM EDS of "*as recovered*" graphite……………………………………..…..S14

Fig. S12. NMR of recovered anode binder from 220023-2-6-120……………………………S15

Fig. S13. SEM/EDS of recovered Cu current collector from Samsung 220023-2-6-120…...S16

Table S1. Details of commercial Samsung 18650 cylindrical cells.

**Battery Type: 2.6A Samsung 18650**	**220018-4-6-109**	**220023-2-4-120**
Total Cycle Numbers	811	1120
Cycling Protocol	40ºC, 2C CCCV	25ºC, 1C CC
Voltage Limits	2.7 to 4.2 V	2.7 to 4.2 V
Average CE (%)	99.977%	99.231%

Cells were cycled between 2017-2019 for various temperature or environemntal condition lifetime testing. Battery packs did not contain information about the chemistry of the cathode and the binder.

Section 1. Parameter Optimization for DMI-based Cathode Delamination

A temperature range of 120–150 °C and the reaction time for 15 minutes to 24 h were explored to determine the experiment setup that suits the best DMI-based delamination condition for cathode active materials. We note that while most home-made cathode composites were dissolved in DMI only after 30 minutes heating at 150 ºC, the process had to be extended to at least 5 h for those cathodes that were harvested from EoL Samsung batteries. Extended time is needed possibly due to differences in cathode manufacturing, or electrochemical treatment. A solid-to liquid (S/L) ratio was optimized to 1:40 g:mL for ease of separation of cathode components, however, a lower S/L ratio is aimed to decrease the solvent cost and investigated in our lab currently. Attempt to reduce the solvent volume for more economical delamination was carried out for EoL Samsung anode materials that results into use of a lower S/L ratio of 1:10 g:mL, described in **Section 3**. All the optimization results are presented in **Table S2**.

Table S2. Summary of optimized experimental parameters for DMI-based cathode delamination.

Cathode Type	Cathode Appearance Before Delamination	Reaction Temperature (ºC)	Reaction Time (h)	Appearance After Delamination	Comment
Homemade NMC composite film (soaked in $LiPF_6$ electrolyte for a week, no electrochemical cycling was performed)	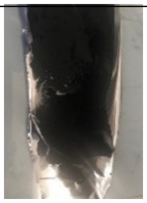	150	0.5	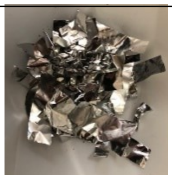 Bare Al substrate, no significant trace of AMs	All these conditions result into successful delamination of active materials.
		140	1		
		130	1		
Samsung Battery 220018-4-6-109	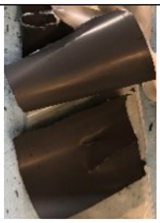	150	24	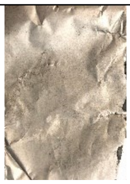 Bare substrate, no significant trace of AMs	Both conditions result into bare Al substrate with no sign of active materials. It suggests successful delamination.
		150	5		
Samsung Battery 220018-4-6-109	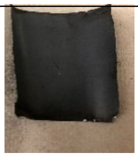	130	24	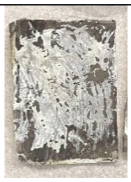 Significant residue of AMs on substrate	Active materials were not delaminated fully.
Samsung Battery 220018-4-6-109	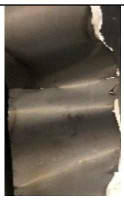	120	24	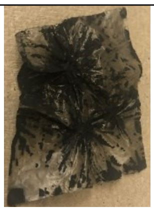 Significant residue of AMs on substrate	Cathode active material delamination was not satisfactory.

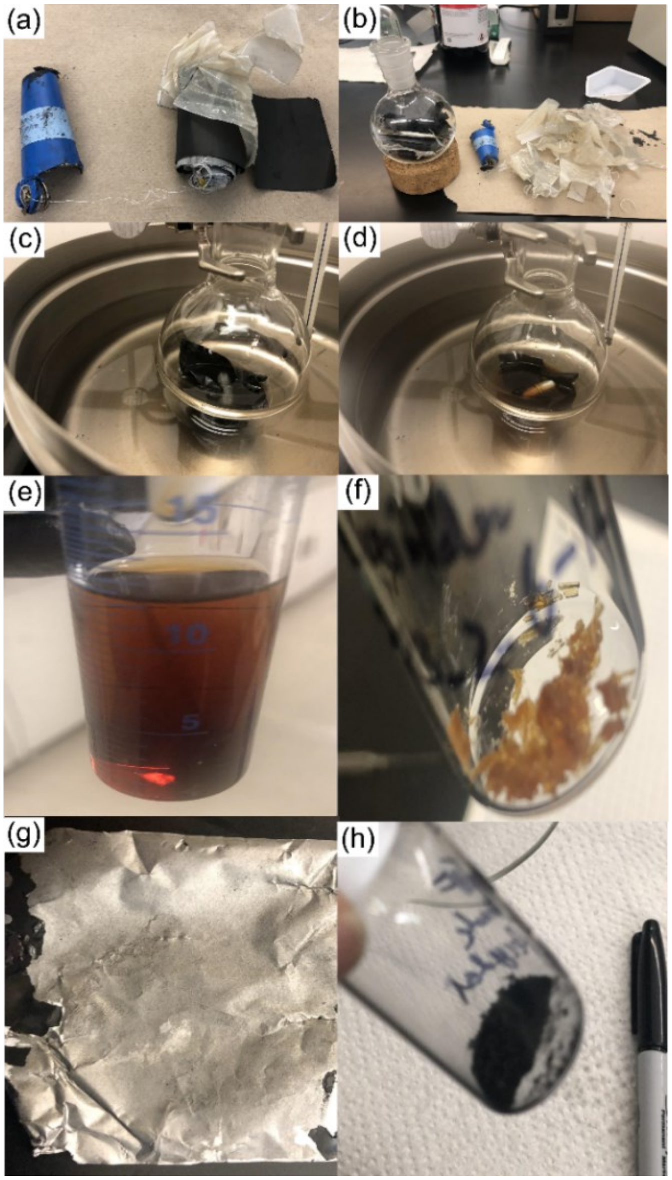

Fig. S1. Cathode film was dismantled from commercial battery Samsung 18650s, Neware ID: 220018-4-6-109 and separated from outer plastic case, anode and separator (a-b). A vacuum flask was equipped with stir bar and filled with 1.095 g cathode and 40 mL DMI (c). Reaction was continued at 150 ºC for 24 hours (it must be noted that the same delamination efficiency found while the reaction was conducted at 150 ºC for 5 hours) (d). A clear dark brown solution was obtained upon filtration and separated from aluminum substrate and cathode AMs (e). Filtrate was precipitated in chilled diethyl ether and 0.029 g yellowish-brown colored binder obtained upon drying under vacuum. O.195 g bare substrate (g) and a mixture of AMs + conductive C of 0.86 g

(h) were found after manual scraping. Obtained cathode AMs was sonicated in hot DMI and washed with diethyl ether to remove residual PVDF and any other organic impurities, step is not shown.

Table S3. Recovery yield of delaminated cathode components from commercial cell 220023-2-4-120 via DMI.

Cathode Mass (g)	Recovered AMs+C (g) after DMI treatment	% Recovery of cathode AMs+C	Recovered binder (g) after DMI treatment	% Recovery of binder	Al + Active Materials (g)
5	2	(40%)[a]	0.06	(40%)[b]	2.8

Masses of each recovered components were measured in analytical balance. [a]% yield of AMs with conductive carbon mixture was calculated with respect to the total mass of cathode, ignoring the mass of binder and substrate as the specific amounts of individual component in original cathode was unknown. Thus % recovery for AMs+conductive C is calculated as: $\% \, Recovery = \left(\frac{Mass \; of \; AMs \; \& \; conductive \; C \; after \; DMI \; treatment}{Total \; cathode \; mass \; 5 \; g}\right) 100$. [b]Assuming this cell contains 3% binder (as typical commercial batteries consist of 2-4% binder) the % recovery was calculated as: $\% \, Recovery = \left(\frac{Mass \; of \; recovered \; binder \; after \; DMI \; treatment}{3\% \; binder \; present \; in \; 5 \; g \; cathode}\right) 100$.

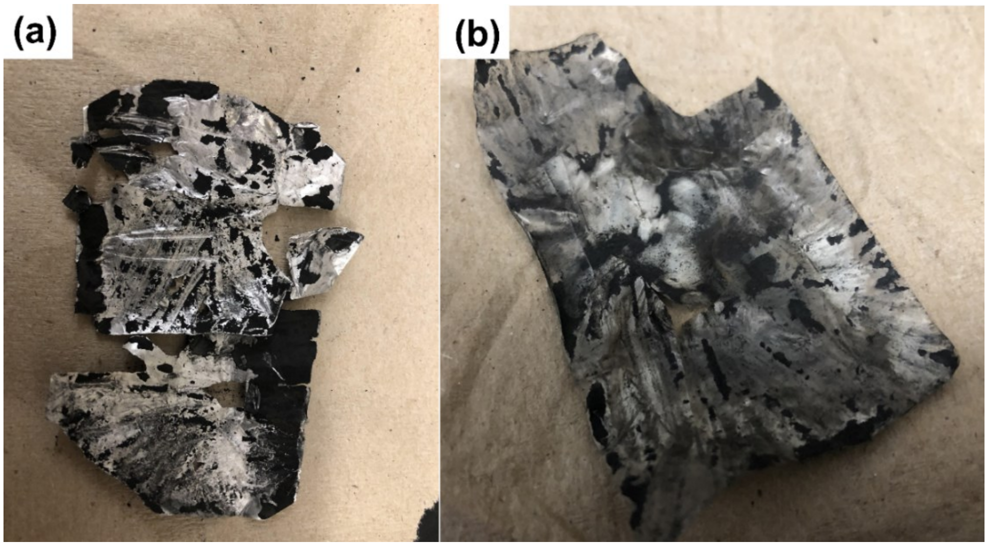

Fig. S2. Recovered Al substrate from EoL Samsung 220023-2-4-120, treated in DMI (a) and NMP (b). Active materials were not fully delaminated in both cases, even after thorough manual scraping. It is most likely due to the extended electrochemical cycling (cycled at 1C for 1120 cycles at 25 ºC prior to disassembly) which result into sticking of active materials in the current collector, decreases solubility of binder in solvent (e.g., DMI, NMP), and prohibits full liberation of active materials.

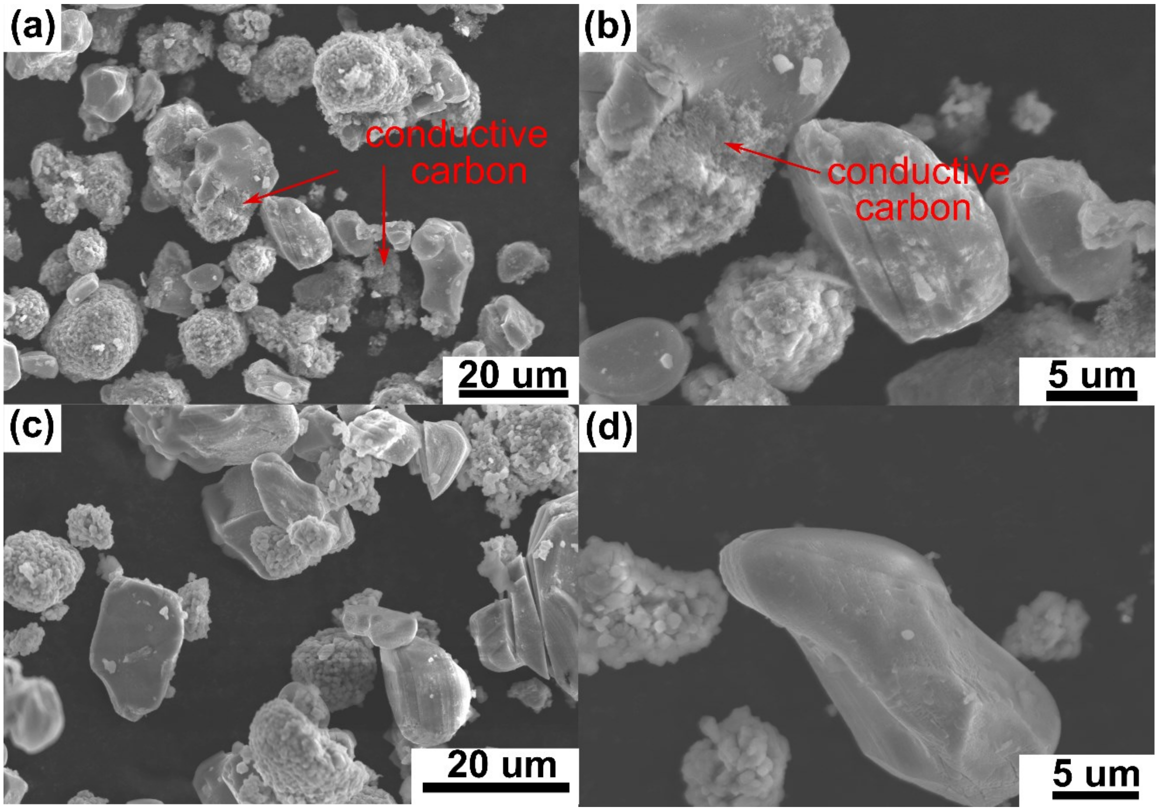

Fig. S3. SEM image of the "*as recovered*" cathode AMs (a-b) showed the presence of conductive carbon, majority of that removed after calcination at 600 ºC in air (c-d).

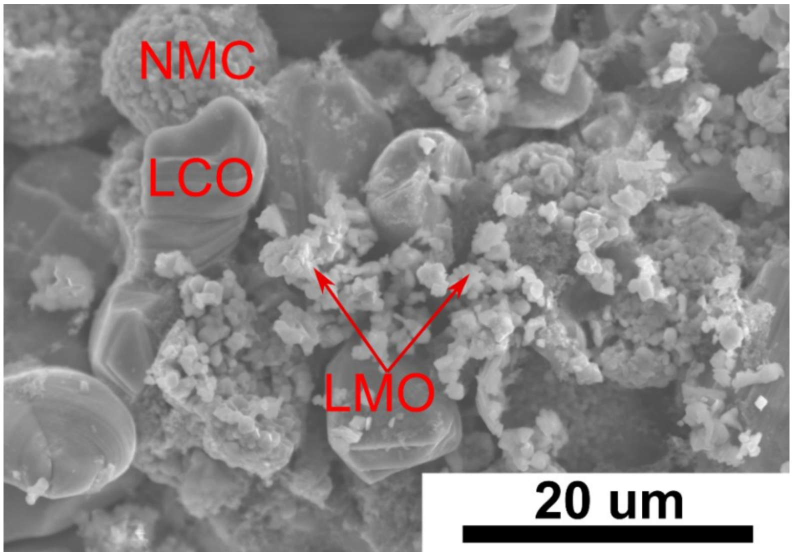

Fig. S4. SEM image of the cathode AMs recovered from Samsung 220018-4-6-109 suggests the electrode is mixed or blended with possible compositions of the LCO, LMO and NMC particles.

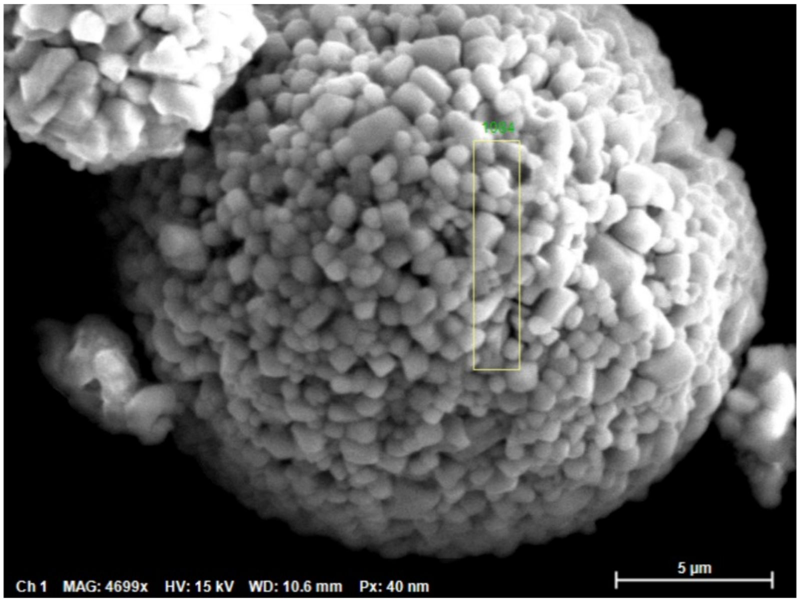

Fig. S5. SEM image of the cathode AMs recovered from Samsung 220018-4-6-109 shows the round-shaped secondary NMC with corresponding primary particles (indicated in box).

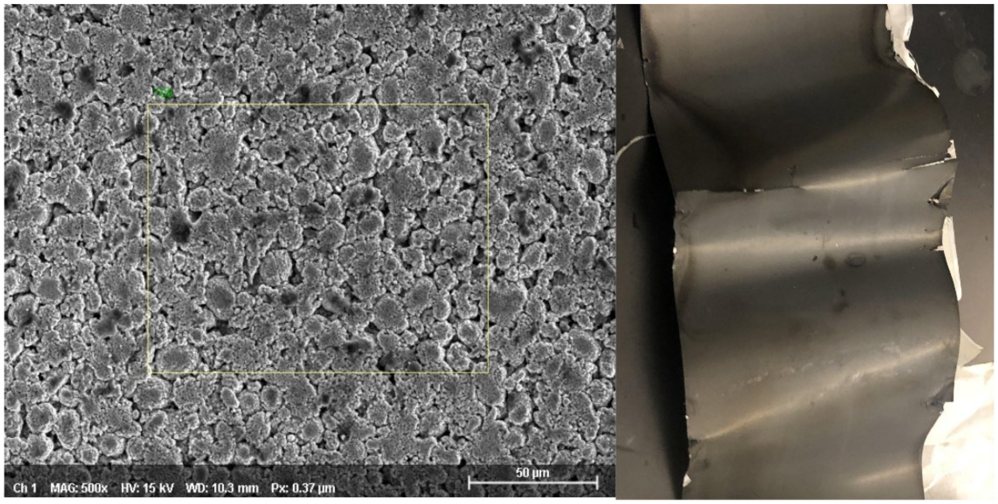

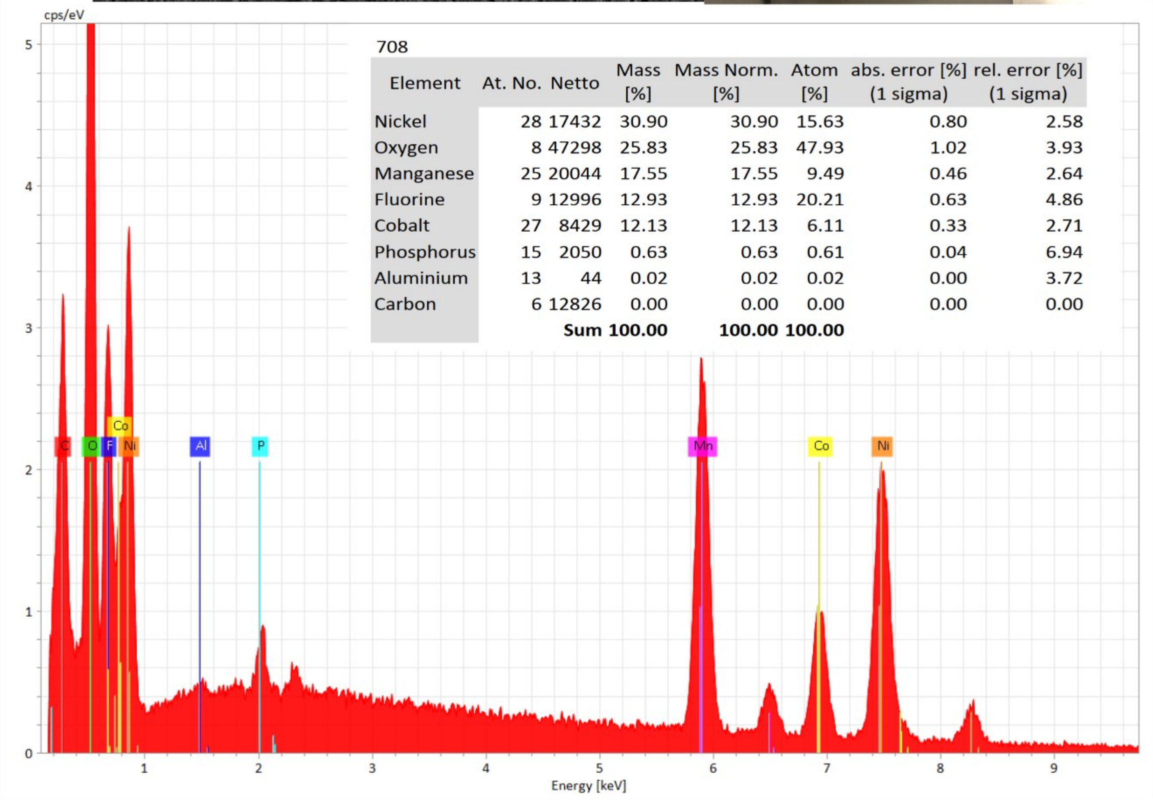

708

Element	At. No.	Netto	Mass [%]	Mass Norm. [%]	Atom [%]	abs. error [%] (1 sigma)	rel. error [%] (1 sigma)
Nickel	28	17432	30.90	30.90	15.63	0.80	2.58
Oxygen	8	47298	25.83	25.83	47.93	1.02	3.93
Manganese	25	20044	17.55	17.55	9.49	0.46	2.64
Fluorine	9	12996	12.93	12.93	20.21	0.63	4.86
Cobalt	27	8429	12.13	12.13	6.11	0.33	2.71
Phosphorus	15	2050	0.63	0.63	0.61	0.04	6.94
Aluminium	13	44	0.02	0.02	0.02	0.00	3.72
Carbon	6	12826	0.00	0.00	0.00	0.00	0.00
		**Sum**	**100.00**	**100.00**	**100.00**		

Fig. S6. SEM image and corresponding EDS analysis of cathode separated from Samsung 220018-4-6-109 showed the presence of metals including Co, Ni, and Mn. Traces of PVDF binder, conductive carbon and Al substrates were also visible. It detected P that suggests the trace presence

of $LiPF_6$ electrolyte which was not removed completely during cathode washing step. A summary of elemental composition is shown in the same figure.

Section 2. Homemade Composite Cathode Film Fabrication and delaminated using DMI.

400 mg of cathode active material NMC111 was hand ground in a mortar and pestle with 50 mg carbon black until a fine powder obtained. Separately, 50 mg of PVDF was dissolved in 60 drops (~1.4 mL) of NMP. NMC111, carbon black and PVDF were combined in a 80 : 10 : 10 ratio to obtain a slurry that was cast onto an Al substrate. Film was dried on a hot plate set to 100 °C for 1 hour and vacuum dried for 12 hours at 80 °C. Dried film was kept in contact of 5 mL $LiPF_6$ electrolyte for a week followed by washing 3 times with 5 mL dimethyl carbonate. Please note that no electrochemical cycling experiments were conducted on these films. Washed and dried films were treated with DMI following the same procedure described in **Fig. S1**. Delamination was conducted at 150 ºC for 30 minutes and % recoveries of each material are presented in **Table S4**. Recovered materials were calcined and analyzed by SEM, EDS and powder XRD, shown in **Fig. S7, Fig. S8** and **Fig. S9**, respectively.

Table S4. Recovery masses and % yields of delaminated cathode components from homemade composite film via DMI.

**Mass of Al in composite film (mg)**	**Mass of NMC111 +C in composite film (mg)**	**Mass of PVDF in composite film (mg)**	**Mass of Recovered Al after DMI treatment (mg)**	**% Recovery for Al**	**Mass of Recovered NMC111+C after DMI treatment (mg)**	**% Recovery for NMC111 +C**	**Mass of Recovered PVDF after DMI treatment (mg)**	**% Recovery for PVDF**
150	360	40	150	100	354	98	31	77

A total mass of composite cathode film (NMC111+C+PVDF+Al) of 550 mg was taken into consideration for cathode delamination experiment. After delamination, all the recovered materials were washed, and vacuum dried for 2 days. Masses of each dried components were measured in analytical balance. % recovery

is calculated as: $\% \, Recovery = (\frac{Mass\ of\ X\ in\ composite\ film}{Mass\ of\ X\ delaminated\ after\ DMI\ treatment})\ 100$, where x is Al/ mixture of NMC111+C/PVDF binder.

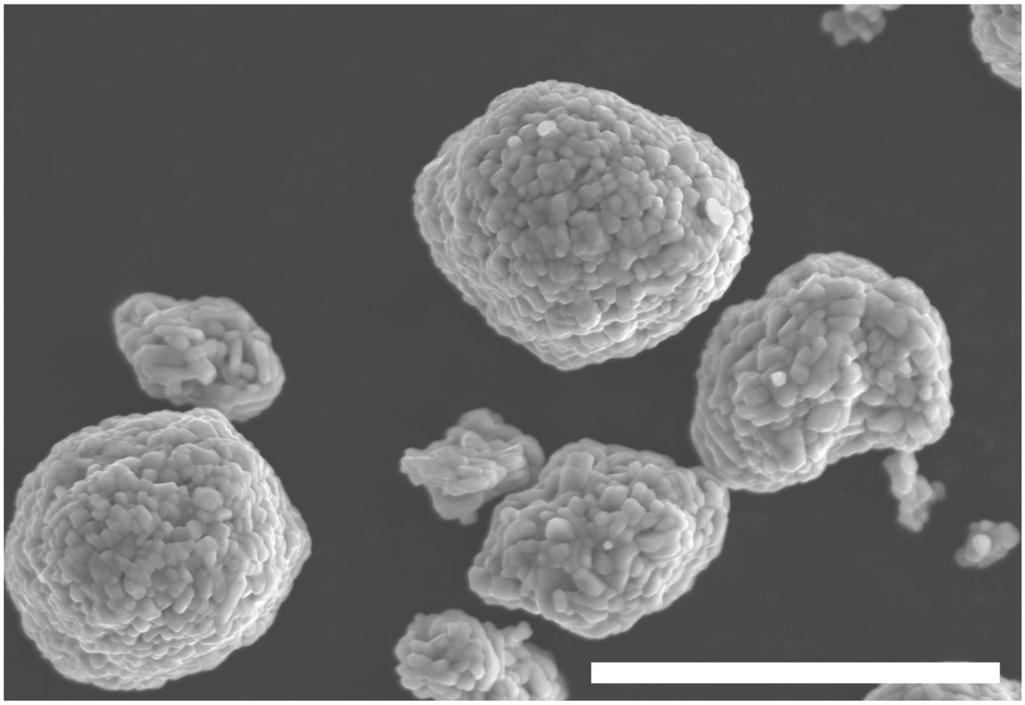

Fig. S7. SEM image of NMC 111 delaminated from homemade film showed that DMI did not jeopardize the NMC surface morphology. Recovered NMC sample was calcined at 600 ^{0}C prior to imaging. Scale bar is set at 20 μm.

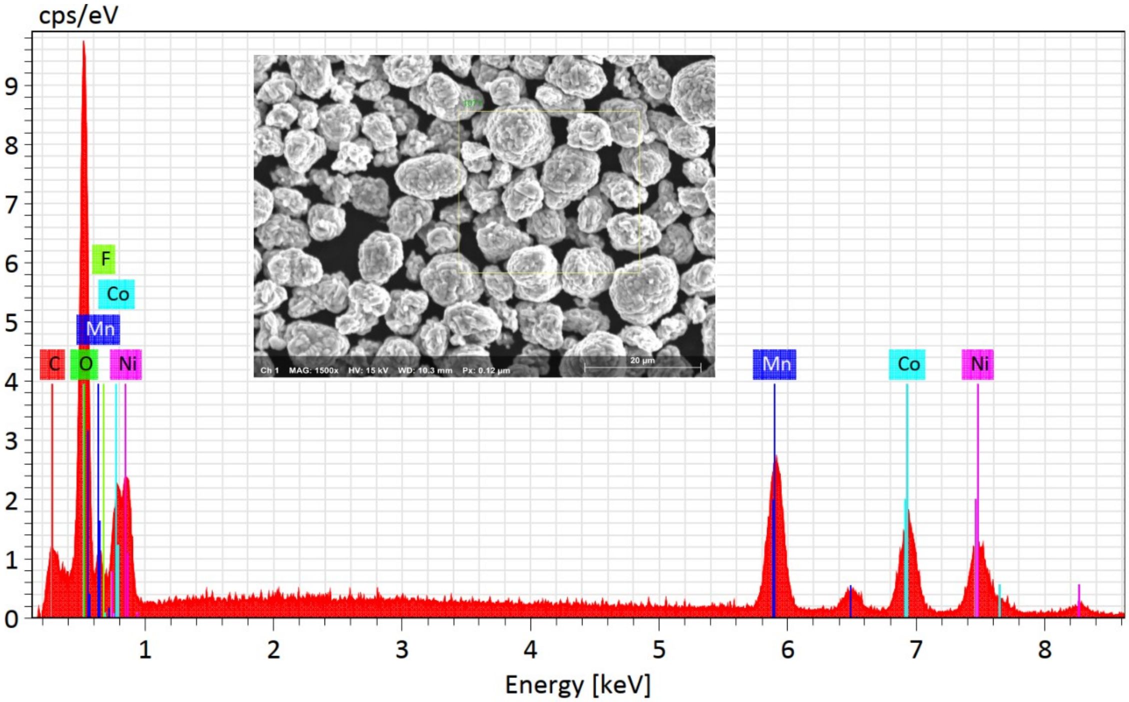

Element	At. No.	Netto	Mass [%]	Mass Norm. [%]	Atom [%]	abs. error [%] (1 sigma)	rel. error [%] (1 sigma)
Nickel	28	12003	25.90	25.90	15.46	0.67	2.60
Cobalt	27	14618	25.64	25.64	15.24	0.67	2.60
Oxygen	8	35847	24.73	24.73	54.16	0.99	3.99
Manganese	25	22540	23.73	23.73	15.13	0.62	2.61
Carbon	6	4607	0.00	0.00	0.00	0.00	0.00
Fluorine	9	0	0.00	0.00	0.00	0.00	4.75
		**Sum**	**100.00**	**100.00**	**100.00**		

Fig. S8. EDS analysis of NMC 111 delaminated from homemade film followed by calcination showed that DMI based methodology did not affect the elemental composition.

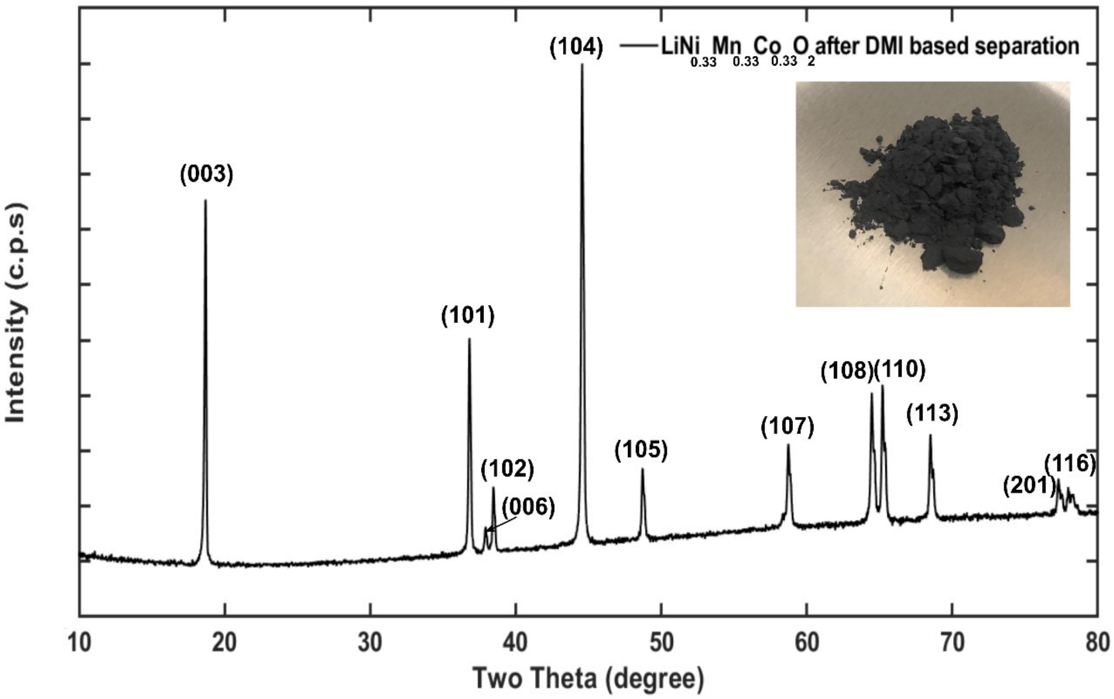

Fig. S9. XRD of NMC 111 delaminated from homemade film. Peak indexed to the R-3m space groups are labeled and showed no trace of secondary phases and other impurities. XRD pattern suggested our developed DMI based cathode delamination process did not alter crystal structures.

Section 3. Lab Scale Anode Delamination for Samsung 220023-2-4-120

Anode delamination was performed following the same procedure described in **Fig. S1**. However, a lower S/L ratio of 1:10 g:mL was used. Also, no prior washing of anode was conducted to reduce the solvent consumption. Briefly, 10.4 g anode material was dispersed in 100 mL DMI and heated at 140 ºC for 3 hours. 6.1 g graphite and conductive carbon mixture, 3.5 g copper (Cu) substrate and 0.2 g organics were recovered. Characterization results of the recovered materials are shown in **Fig. S10- S13**.

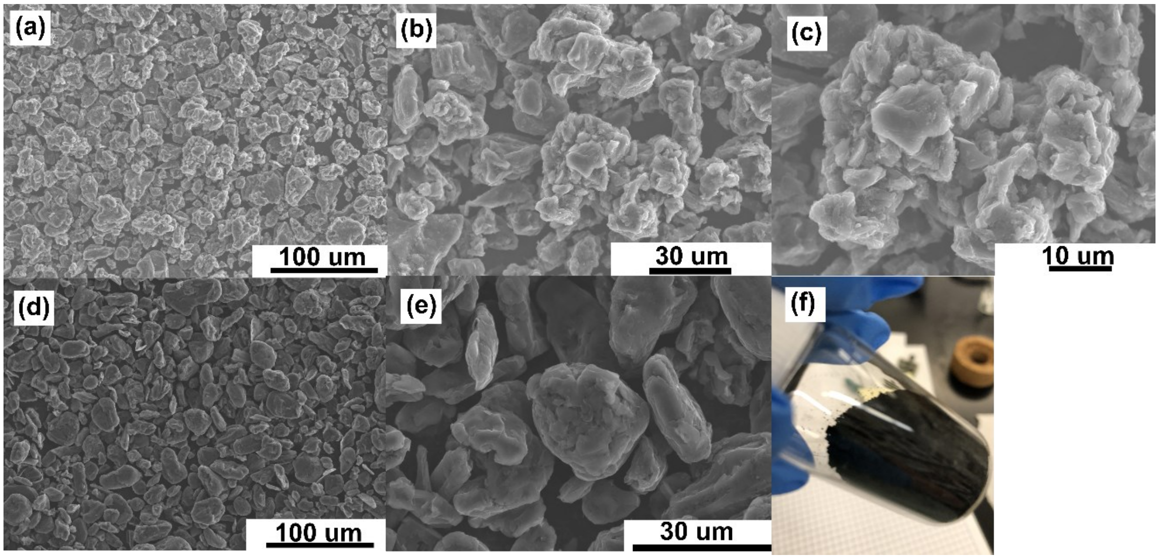

Fig. S10. SEM micrograph of the “*as recovered*” graphite and conductive carbon mixture (a-c) shows agglomeration phenomena compared to commercially purchased graphite (d-e). Agglomeration texture possibly arises due to the presence of metal impurities and organic binder. Picture of recovered graphite is shown in (f).

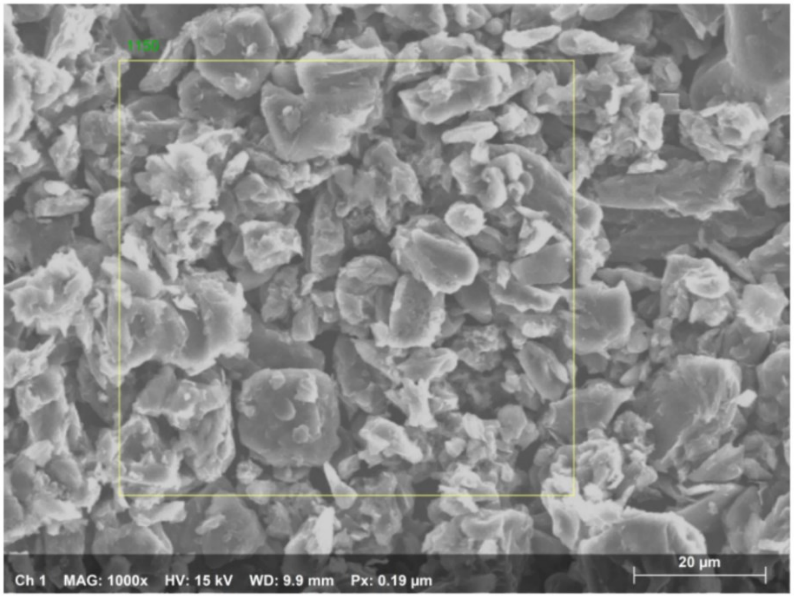

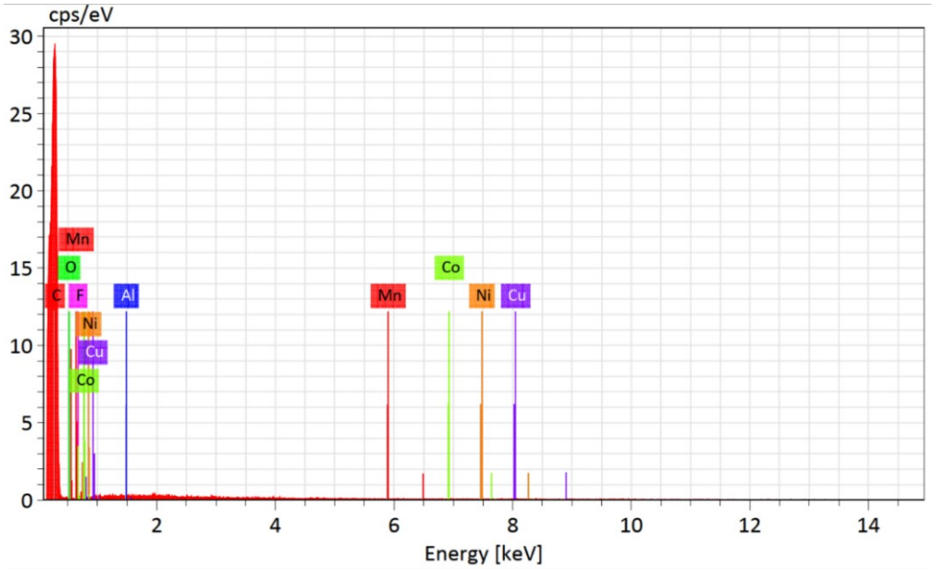

Element	At. No.	Netto	Mass [%]	Mass Norm. [%]	Atom [%]	Comp.	Sto. [%]	Sto. Norm. [%]	abs. error [%] (1 sigma)
Carbon	6	126236	99.27	99.27	99.85		99.27	99.27	3.84
Copper	29	178	0.70	0.70	0.13		0.70	0.70	0.04
Aluminium	13	34	0.01	0.01	0.01	Al2O3	0.02	0.02	0.00
Cobalt	27	5	0.01	0.01	0.00		0.01	0.01	0.00
Oxygen	8	542	0.01	0.01	0.01		0.00	0.00	0.00
Fluorine	9	0	0.00	0.00	0.00		0.00	0.00	0.00
Nickel	28	0	0.00	0.00	0.00		0.00	0.00	0.00
Manganese	25	0	0.00	0.00	0.00		0.00	0.00	0.00
		**Sum**	**100.00**	**100.00**	**100.00**		**100.00**	**100.00**	

Fig. S11. SEM EDS of "*as recovered*" graphite from Samsung 220023-2-4-120 demonstrates trace presence of metal impurities.

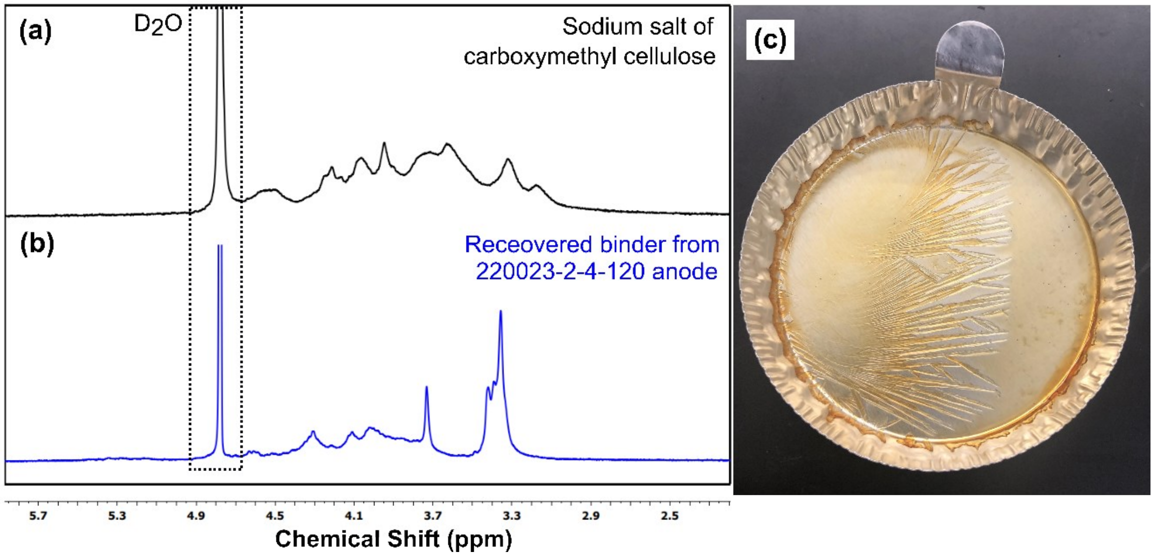

Fig. S12. ^{1}H NMR spectrum of recovered anode binder in D_2O (a), compared with commercially purchased sodium salt of carboxymethyl cellulose (CMC) (b), that is typically used as anode binder. However, the NMR shows many unidentified peaks, possibly arises from electrolyte additives and other impurities that generated during extended electrochemical cycling. The recovered needle-like crystalline organic binder is shown in (c).

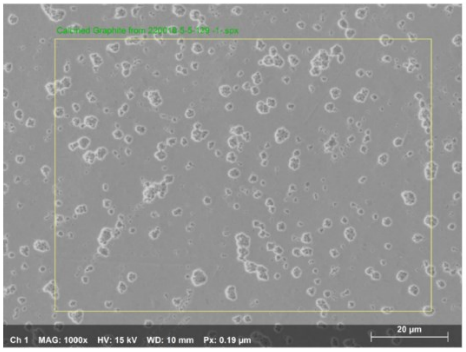

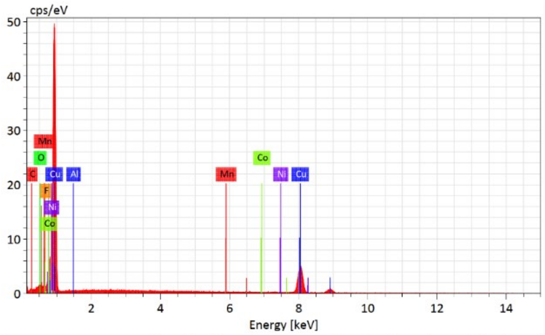

Element	At. No.	Netto	Mass [%]	Mass Norm. [%]	Atom [%]	Comp.	Sto. [%]	Sto. Norm. [%]	abs. error [%] (1 sigma)	rel. error [%] (1 sigma)
Copper	29	53625	94.21	94.21	77.28		94.21	94.21	2.38	2.53
Carbon	6	2835	4.80	4.80	20.84		4.80	4.80	0.27	5.71
Fluorine	9	1552	0.52	0.52	1.43		0.52	0.52	0.04	8.56
Nickel	28	298	0.37	0.37	0.33		0.37	0.37	0.03	9.33
Cobalt	27	115	0.08	0.08	0.07		0.08	0.08	0.03	32.45
Aluminium	13	33	0.01	0.01	0.02	Al2O3	0.02	0.02	0.00	4.10
Oxygen	8	4604	0.01	0.01	0.03		0.00	0.00	0.00	4.10
Manganese	25	0	0.00	0.00	0.00		0.00	0.00	0.00	2.50
		**Sum**	**100.00**	**100.00**	**100.00**		**100.00**	**100.00**		

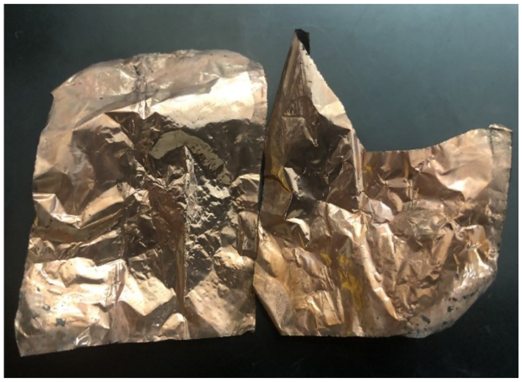

Fig. S13. SEM surface image and EDS analysis of recovered Cu foil. Trace amount of conductive carbon and active materials (Ni, Co) were found which could be delaminated completely after sonication. A trace of F residue (0.5%) was found in EDS. Please note that PVDF was not recognized as anode binder (shown in NMR, **Fig. S12**). Thus, the F residue possibly comes from the $LiPF_6$ electrolyte which was not eliminated completely as no prior washing step was performed before DMI-based delamination treatment.
